# Isolation and characterization of cadmium-resistant *Bacillus cereus* strains from Cd-contaminated mining areas for potential bioremediation applications

**DOI:** 10.3389/fmicb.2025.1550830

**Published:** 2025-02-12

**Authors:** Bixia Liang, Yimeng Feng, Xiyue Ji, Chune Li, Qian Li, Zhenshun Zeng, Yuqi Wang

**Affiliations:** School of Environmental Science and Engineering, Guangzhou University, Guangzhou, China

**Keywords:** *Bacillus cereus*, Cd resistance, bioremediation, siderophore production, heavy metal tolerance, Cd adsorption

## Abstract

Cadmium (Cd) is a naturally occurring heavy metal found in the soil. However, its concentrations can be substantially increased by anthropogenic activities, presenting considerable environmental challenges. One effective remediation strategy is soil bioremediation, which employs indigenous bacteria to mitigate contamination. This study aimed to identify Cd-resistant bacteria and assess their potential for bioremediating Cd-contaminated soil. Two Cd-resistant bacterial strains, designated C9 and C27, were isolated from Cd-contaminated soil at concentrations ranging from 100 to 500 mg/L. Morphological analysis and 16S rDNA sequencing identified both strains as *Bacillus cereus*. The strains’ capacity to adsorb and remove Cd from solutions was assessed, as well as their resistance to other heavy metals, including Zinc (Zn) and Thallium (Tl). Optimal Cd adsorption was observed at 36 h for strain C9 and at 48 h for strain C27, with maximum removal rates achieved at a Cd concentration of 70 μM. Both strains demonstrated substantial resistance to heavy metals in the order Zn > Cd > Tl on solid media. Additionally, they exhibited strong salt tolerance, starch hydrolysis, citrate utilization, and ammonia production capabilities. Notably, both strains produced significantly higher levels of siderophores compared to the model bacterium *Bacillus subtilis* 3,610, with strain C9 exhibiting superior siderophore production. This enhanced siderophore activity is hypothesized to contribute to Cd resistance. Collectively, these findings suggest that strains C9 and C27 have significant potential for the bioremediation of Cd-contaminated environments. Future research will focus on elucidating the molecular mechanisms underlying heavy-metal resistance and optimizing their application in large-scale bioremediation strategies.

## 1 Introduction

Cadmium (Cd) is a highly toxic heavy metal that poses a significant threat to both environmental and human health. The International Agency for Research on Cancer (IARC) classifies Cd as a Group 1 carcinogen, associating it with an increased risk of prostate, kidney, and lung cancers, as well as chromosomal aberrations and other health issues (Nogawa et al., 2022; Yang et al., 2022). Nishijo et al. (2004) conducted a prospective epidemiological survey on the population in Kakehashi, Japan, which is contaminated by Cd, for 15 years. The results found that individuals living in Cd-contaminated areas had a higher probability of death from malignant tumors compared to those living in non-Cd-contaminated areas. Industrial processes such as mining, smelting, electroplating, along with agricultural practices involving phosphate fertilizers and sewage sludge application, are the main sources of cadmium pollution in the environment (Dong et al., 2022; Feng et al., 2022; Huang et al., 2022; Khan et al., 2022). The soil environmental issues triggered by Cd have progressively deepened, attracting considerable attention around the world (Shi et al., 2022). Therefore, the remediation of Cd-contaminated soil is urgent. Remediation methods for heavy metal-contaminated soil can be categorized into physical remediation, chemical remediation and microbial remediation techniques (Shuang et al., 2021; Sajjad et al., 2024). Microbial remediation, an advanced remediation technology developed under the guidance of modern scientific and technological advancements, is distinguished by its extremely low cost, easy operation and non-pollution (Sajjad et al., 2019; Dhaliwal et al., 2020; Peng et al., 2018). The persistent nature of cadmium, coupled with its inability to biodegrade, results in its retention in soil systems. Subsequently, plants uptake this toxic metal from contaminated soils, facilitating its entry and accumulation throughout the food chain (Benavides et al., 2005).

Recently, the environmental pollution problem caused by Cd has gradually worsened (Shi et al., 2022). The vast majority of Cd intake in the human body is ingested through grains and vegetables (Sindhu et al., 2022; Li et al., 2022). Therefore, how to efficiently reduce the Cd content in soil has become a widespread concern. Currently, there have been numerous studies on the removal of Cd from Cd-polluted soil using microbial techniques. The remediation of heavy metal contamination through traditional approaches such as excavation, solidification, and chemical treatments presents significant challenges, including substantial financial costs, intensive labor requirements, and detrimental environmental consequences (Sajjad et al., 2020; Dhaliwal et al., 2020). In response to these limitations, microbial-based remediation strategies have gained attention as an environmentally sustainable and economically viable alternative. Junpradit et al. (2021) found that strains capable of stimulating the growth and Cd uptake in *C. comosum* could remove Cd from aqueous solutions and survive in Cd-contaminated soil. Schütze et al. (2014) reported that microbial-assisted phytoremediation had a positive effect on phytoremediation performance of heavy metal-contaminated soils. The isolation and screening experiment conducted by Khanthom et al. (2021) showed that *E. cloacae* CdRR1 could promote shoot and root elongation. These beneficial microorganisms exhibit remarkable capabilities in heavy metal detoxification through multiple cellular processes, including adsorption, precipitation, and binding mechanisms. Additionally, they contribute to ecosystem health by facilitating plant growth through enhanced nutrient uptake and improved soil quality (Verma and Kuila, 2019; Priyadarshini et al., 2021).

However, the mechanism of Cd resistance in Cd-resistant strains and the mechanism for removing Cd from soil are less studied. It has been found that heavy metal-resistant microorganisms can reduce the bioavailability of Cd through adsorption, precipitation, and binding fixation to alleviate Cd contamination. They can also promote the uptake of Cd^2+^ by hyperaccumulator plants through dissolving and activating processes to remove Cd from the soil (Carlot et al., 2010). Consequently, isolating heavy metal-resistant bacteria in soil is important for advancing the application of microorganisms in environmental remediation and protection. Despite the promising potential of microbial remediation, significant research gaps persist in elucidating the fundamental mechanisms of microbial adaptation to cadmium stress and optimizing their implementation in practical soil restoration efforts. The *Bacillus* genus is renowned for its adaptability to extreme conditions and its capacity to resist and detoxify heavy metals (Saxena et al., 2020). Species such as *Bacillus cereus* have been widely investigated for their bioremediation potential, attributed to their production of biofilms, siderophores, and extracellular enzymes, which improve metal adsorption and resistance (Sajjad et al., 2020; Mishra et al., 2022; Zada et al., 2024). Recent research highlights the crucial role of siderophores in alleviating metal toxicity by chelating metal ions and promoting their uptake or immobilization (Roy et al., 2022). In addition, Chen et al. (2019) discovered that the differential gene expression enriched in the KEGG pathway, which mitigates Cd toxicity, is associated with starch and sucrose metabolism. This metabolic process reduces Cd toxicity by enhancing the activity of antioxidant and transport systems. Sucrose and starch metabolism also activate the expression of related genes and the phosphorylation of proteins, thus alleviating the damage caused by Cd to plants (He et al., 2021). Cd and citric acid can also prevent plant toxicity by forming thermodynamically assisted complexes. These complexes dissociate Cd from the weak bonds adsorbed in the soil particle structure, facilitating the removal of Cd from soil cation exchange sites (Adeniji et al., 2010). Citric acid also plays a crucial role in reducing the effectiveness of Cd on plants. It forms chelates that prevent the transport of Cd^2+^ in the cytoplasm, thereby impeding its entry into plant roots in the free form (Mench and Martin, 1991; Li et al., 2014).

This study seeks to address the urgent need for effective cadmium (Cd) remediation by isolating and characterizing Cd-resistant bacterial strains from contaminated soils. Two strains, identified as *Bacillus cereus* (C9 and C27), were examined for their Cd adsorption and removal capabilities under different environmental conditions. Their resistance to other heavy metals, including zinc (Zn) and thallium (Tl), was also evaluated, alongside physiological traits such as salt tolerance, starch hydrolysis, citrate utilization, ammonia production, and siderophore production. By uncovering the factors underlying their Cd resistance, this research provides a basis for developing sustainable microbial remediation strategies and enhances understanding of *Bacillus cereus* as a potential solution to heavy metal contamination in agricultural and industrial soils.

## 2 Materials and methods

### 2.1 Soil samples and experimental strains

Soil samples (collected in Lanmuchang, Guizhou) were collected from a mining area with Cadmium (Cd) contamination exceeding regulatory limits (100–500 mg/L). The samples were transported in sterile containers and stored at 4°C prior to processing. Cd-resistant bacterial strains were isolated through serial dilution and screening with increasing concentrations of Cd in the culture media. The model bacterium *Bacillus subtilis* 3,610 was used as a reference strain for comparative analyses (Sonenshein et al., 2001).

### 2.2 Isolation of Cd-resistant bacteria

Approximately 2 g of soil sample was suspended in 10 mL of sterilized water and agitated for 30 min to dislodge bacteria. A 50 μL aliquot of the suspension was spread on Luria-Bertani (LB) agar medium and incubated at 30°C for 24 h. The colonies were then transferred to LB agar containing 100–500 mg/L Cd^2+^ and incubated at 30°C for 72 h to assess Cd tolerance. Highly resistant colonies were purified by repeated streaking on Cd-containing LB agar to obtain pure cultures.

### 2.3 Morphological characterization and molecular identification

Colony morphology, including size, shape and color was observed on LB agar plates. The Gram staining and spore staining were performed on bacteria in the exponential growth phase and visualized under a light microscope to observe the gram reaction and the cell spores.

Genomic DNA was extracted using a commercial bacterial DNA kit (Omega, USA). The 16S rDNA region was amplified using universal primers (27F and 1492R) under the following PCR conditions: initial denaturation at 94°C for 3.5 min, followed by 30 cycles of denaturation (94°C for 30 s), annealing (55°C for 30 s), and extension (72°C for 1.5 min). PCR products were sequenced and the 16S rDNA sequence was subjected to BLAST search in the NCBI database. The phylogenetic tree was constructed using the neighbor-joining method to confirm the identity of the strains.

### 2.4 Cd adsorption and removal

The Cd adsorption and removal capacities of the bacterial strains were evaluated using inductively coupled plasma mass spectrometry (ICP-MS) (Shimadzu, Japan). This experiment aimed to assess the Cd uptake by two strains under varying metal concentrations, pH levels, and time conditions. Each experimental group was tested in triplicate. Prior to the experiments, the strains were cultured in LB liquid medium and activated. Their optical density (OD) was measured at 600 nm using a UV–visible spectrophotometer.

### 2.5 Effect of metal concentration, pH and incubation time on Cd adsorption

To determine the effect of the exposed Cd concentration on bacterial adsorption, the bacterial strains were inoculated into tubes, each containing Cd^2+^ at a final concentration of 40, 50, 60, 70, and 90 μM (4.5 mg/L, 5.62 mg/L, 6.74 mg/L, 7.87 mg/L, and 10.12 mg/L). These tubes were then incubated in a constant-temperature shaker at 30°C and 150 rpm for 24 h to facilitate adsorption. To determine the effect of pH on Cd adsorption, the strains were inoculated into tubes containing LB liquid medium adjusted to various pH levels (4, 4.5, 5, 6, and 7), with Cd^2+^ added to achieve a final concentration of 50 μM. To determine the effect of incubation time on Cd adsorption, the strains were inoculated into tubes containing LB liquid medium with a Cd^2+^ concentration of 50 μM. These tubes were incubated in a constant-temperature shaker at 30°C and 150 rpm, and the adsorption reactions were allowed to proceed for 24, 28, 32, 36, and 48 h.

After the adsorption reactions, the supernatant was collected, filtered, and diluted with 5% nitric acid for subsequent analysis. The dry weight of the bacterial samples was measured, and 3 mL of 2 N HCl was added to each sample for digestion over 24 h. The Cd adsorption and removal rates (ηCd) of the bacterial strains were determined by ICP-MS. The adsorption rate and removal efficiency were calculated using the following formulas:


(1) $$\text{Adsorption}\;\left(Cd\right)=C\times V/M$$

(2) $$\eta_{Cd}=\left[\left(a-b\right)/a\right]\times 100\%$$

where, C is the bacterial adsorption Cd content (mg/L), V is the liquid volume of the digestion solution (L), M is the bacterial dry weight (g), a is the initial Cd concentration (mg/L), and b is the residual Cd concentration (mg/L).

### 2.6 Resistance to other heavy metals

Minimum inhibitory concentrations (MIC) of Cd, Zn, and Tl were determined by streaking cultures on LB agar plates containing increasing concentrations of the metals (100–500 mg/L Cd^2+^ supplied as CdCl_2_, 600–1,600 mg/L Zn^2+^ supplied as ZnCl_2_, and 200–500 mg/L Tl^+^ supplied as TlNO_3_). The plates were incubated at 30°C for 48 h, and the MIC was recorded as the lowest concentration that inhibited visible growth.

### 2.7 Physiological and biochemical characterization

#### 2.7.1 Indole-3-acetic acid (IAA) production

The strains were inoculated into 10 mL of LB liquid medium containing L-tryptophan and incubated overnight at 30°C in a shaking incubator at 240 rpm. After centrifugation, the supernatant was mixed with the Salkowski reagent and stored in the dark for 30 min. The same procedure was performed using ultrapure water as a blank control. The absorbance of both the samples and blank control was measured at 530 nm using a UV spectrophotometer. An IAA standard curve was constructed using IAA standard solution, and each experiment was performed in triplicate.

#### 2.7.2 Siderophore production

Siderophore production (iron carriers) was assessed using the CAS plate coverage method (Shi et al., 2013). Briefly, the strains were inoculated into liquid LB medium and incubated in a biological shaker at 30°C with continuous shaking at 240 rpm for 48 h. After incubation, the bacterial cultures were diluted 10-fold with ultrapure water to prepare a bacterial suspension. The diluted suspensions were subsequently plated onto LB solid medium and incubated at a constant temperature for 24 h. To detect siderophore production, sterilized modified CAS detection medium was added to the plates. The presence of an orange-yellow halo surrounding the colonies in the upper layer of the CAS detection medium indicated the production of siderophores.

To quantify the siderophore activity, the strains were inoculated into MSA liquid medium and cultured in a constant-temperature shaker for approximately 48 h, and then 1 mL of supernatant was collected from each culture and mixed with 1 mL of CAS assay solution. After 1 h, the absorbance was measured at 630 nm, with sterile MSA liquid medium serving as the control. The siderophore activity (siderophore unit, SU) was calculated using the following formula:


(3) $$SU=\frac{Ar-As}{Ar}\times 100\%$$

Where As represents the absorbance value of the strain culture, and Ar represents the absorbance value of the control.

#### 2.7.3 Biofilm formation analysis

The overnight cultures of two Cd-resistant strains (C9 and C27) and the model bacterium 3,610 were diluted to an initial turbidity at 600 nm of 0.4. Subsequently, 8 μL of the diluted bacterial suspension was inoculated onto LBGM (Luria-Bertani Glucose Medium) solid medium, and 5 mL of the LBGM liquid medium was inoculated with the bacteria cultures at a final inoculum volume of 2%. The samples were incubated in a constant-temperature incubator at 30°C for 24, 48, and 72 h. The surface of the liquid medium was examined for the presence of pellicle, while colonization on the surface of the solid medium was also observed.

#### 2.7.4 Salt tolerance

The overnight cultures of two strains (C9, C27) and the model bacterium (3610) were diluted to an initial turbidity at 600 nm of 0.4. Subsequently, 8 μL of each diluted bacterial suspension was inoculated onto the LB solid media containing 5, 7, 9, 11, and 13% NaCl. The plates were incubated at 30°C for 24 h, after which bacterial growth was observed to assess salt tolerance.

#### 2.7.5 Starch hydrolysis experiment

The strains were inoculated onto starch agar medium plates and incubated at a constant temperature of 30°C for 24 h. After incubation, a small amount of iodine solution was added to each plate. The plates were gently rotated to ensure the iodine solution was evenly distributed across the agar surface. The ability of the strains to hydrolyze starch was determined by the presence of a colorless, transparent halo surrounding the colonies. The formation of such a halo indicated that the strains could hydrolyze starch.

#### 2.7.6 Citrate utilization experiment

The strains were inoculated onto citrate solid medium and incubated in a constant-temperature incubator at 30°C for 24–48 h. The color change of the medium was observed under aseptic conditions. If the medium turned blue, it indicated that the strains were capable of utilizing citrate as a carbon source. Conversely, if no color change occurred, it suggested the strains could not utilize citrate.

#### 2.7.7 Ammonia production experiment

The strains were cultured in 10 mL of sterile peptone water liquid medium and incubated in a shaker at 30°C for 48–52 h. Sterile peptone water medium was used as a blank control. The turbidity of the tubes was observed to assess whether the strains exhibited nitrogen-fixing properties. If turbidity was present, it suggested the strains had nitrogen-fixing capabilities. Additionally, 1 mL of Nessler’s reagent was added to each tube. A color change from turbidity to yellow indicated the production of ammonia (NH_3_) (Kumar et al., 2022).

## 3 Results

### 3.1 Isolation and identification of Cd-resistant bacteria

**Figure 1:**
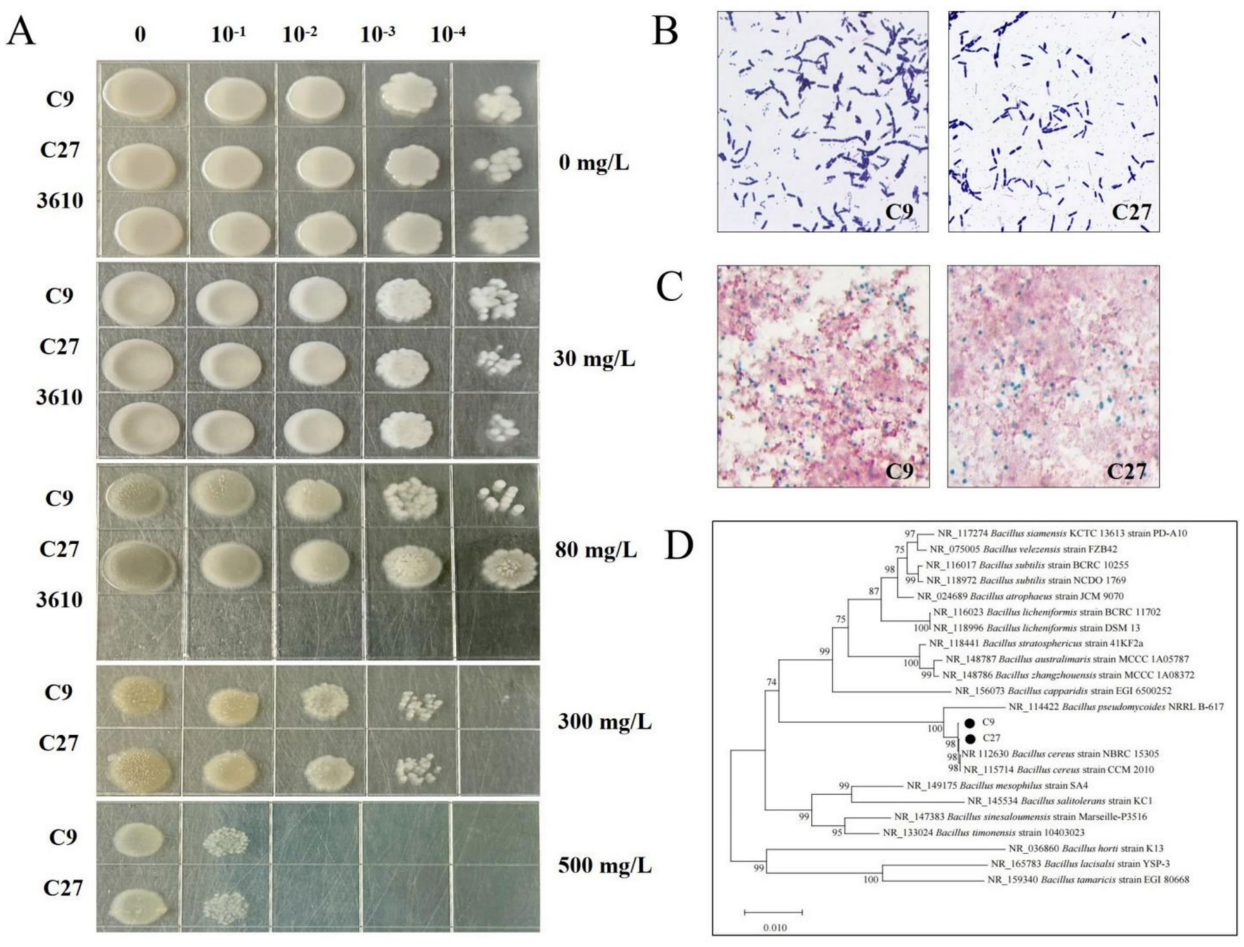
**(A)** The growth of Cd-resistant strains C9, C27, and model bacterium 3,610 at various Cd^2+^ concentrations. **(B)** Gram staining results of Cd-resistant strains C9 and C27. **(C)** Spore staining results of Cd-resistant strains C9 and C27. **(D)** Phylogenetic tree of Cd-resistant strains C9 and C27.

A total of 36 individual Cd-Resistant colonies were isolated from soil contaminated with Cadmium. These isolates were subjected to serial subculturing on plates containing 200–500 mg/L of Cd, which led to the isolation of 11 strains with superior Cd resistance. Among these, two strains, designated as C9 and C27, were selected for subsequent experiments. To compare and verify the Cd tolerance of strains C9, C27, and the model bacterium 3,610, their growth was assessed at Cd^2+^ concentrations of 0, 30, and 80 mg/L. At a Cd^2+^ concentration of 80 mg/L, the model bacterium 3,610 exhibited minimal growth, while strains C9 and C27 displayed normal growth, demonstrating that these two strains possess significantly higher Cd resistance than the model strain 3,610 (Figure 1A). Morphological observation showed that the colonies of strain C9 were white, round, opaque, and had a moist, smooth surface. In contrast, the colonies of strain C27 were also white, round, opaque, but with a dry and smooth surface (Figure 1A). Additionally, Gram staining and spore staining assays revealed that both strains C9 and C27 are Gram-positive and bacillus-forming bacteria (Figures 1B,C).

Genomic DNA extracted from the strains C9 and C27 was used as the PCR templates to amplify the 16S rDNA genes, respectively. The amplified products were then subjected to electrophoresis (Supplementary Figure S1). The electrophoresis analysis revealed that the length of the PCR-amplified fragments for strains C9 and C27 ranged between 1,000 and 2000 bp. Gene sequence homology analysis was conducted using the NCBI database, and the results showed that the 16S rDNA gene sequences of strains C9 and C27 were 99.85% homologous to those of *Bacillus cereus* strain CCM 2010 and *Bacillus cereus* strain NBRC 15305, respectively. Based on the morphological, physiological, and biochemical characteristics, as well as the 16S rDNA sequence analysis, it was concluded that strains C9 and C27 are likely to belong to *Bacillus cereus*. The Neighbor-Joining method was used to construct the phylogenetic tree (Figure 1D). The tree showed that strains C9 and C27 clustered on the same branch as *Bacillus cereus* strain CCM 2010 and *Bacillus cereus* strain NBRC 15305, further supporting the conclusion that strains C9 and C27 belong to *Bacillus cereus*.

### 3.2 Adsorption and removal of Cd

#### 3.2.1 Effect of metal concentration

**Figure 2:**
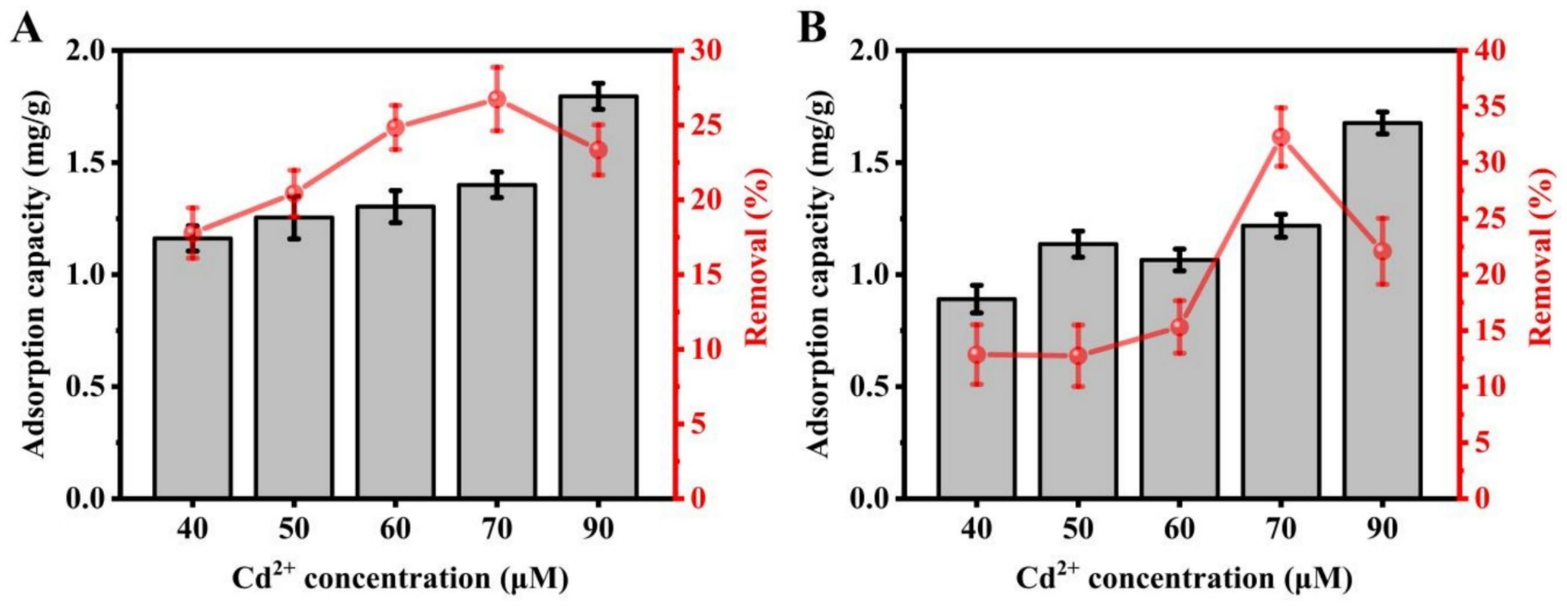
Effect of metal concentration on Cd uptake and removal by strains C9 and C27. **(A)** Strain C9, **(B)** Strain C27.

Cd^2+^ concentrations of 40, 50, 60, 70, and 90 μM were used to analyze the effect on the Cd^2+^ adsorption by the C9 and C27 strains. The ICP-MS assay results showed that strain C9 adsorbed Cd^2+^ in amounts of 1.16 mg/g, 1.25 mg/g, 1.30 mg/g, 1.40 mg/g, and 1.80 mg/g, respectively, corresponding to removal rates of 17.78, 20.43, 24.84, 26.75, and 23.34% (Figure 2). Similarly, the amounts of Cd^2+^ adsorbed by strain C27 were 0.89 mg/g, 1.14 mg/g, 1.07 mg/g, 1.22 mg/g, and 1.68 mg/g, with removal rates of 12.86, 12.75, 15.32, 32.27, and 22.08%, respectively (Figure 2). The adsorption of Cd^2+^ by both strains generally increased with rising metal concentrations. Both strains exhibited an increased removal rate as the Cd^2+^ concentration was raised up to 70 μM. However, the removal rate decreased to 23.34% at a Cd^2+^ concentration of 90 μM. In summary, the optimal initial concentration of Cd^2+^ for adsorption by strains C9 and C27 appeared to be 70 μM, with 90 μM possibly being effective for strain C9.

##### 3.2.1.1 Effect of pH

**Figure 3:**
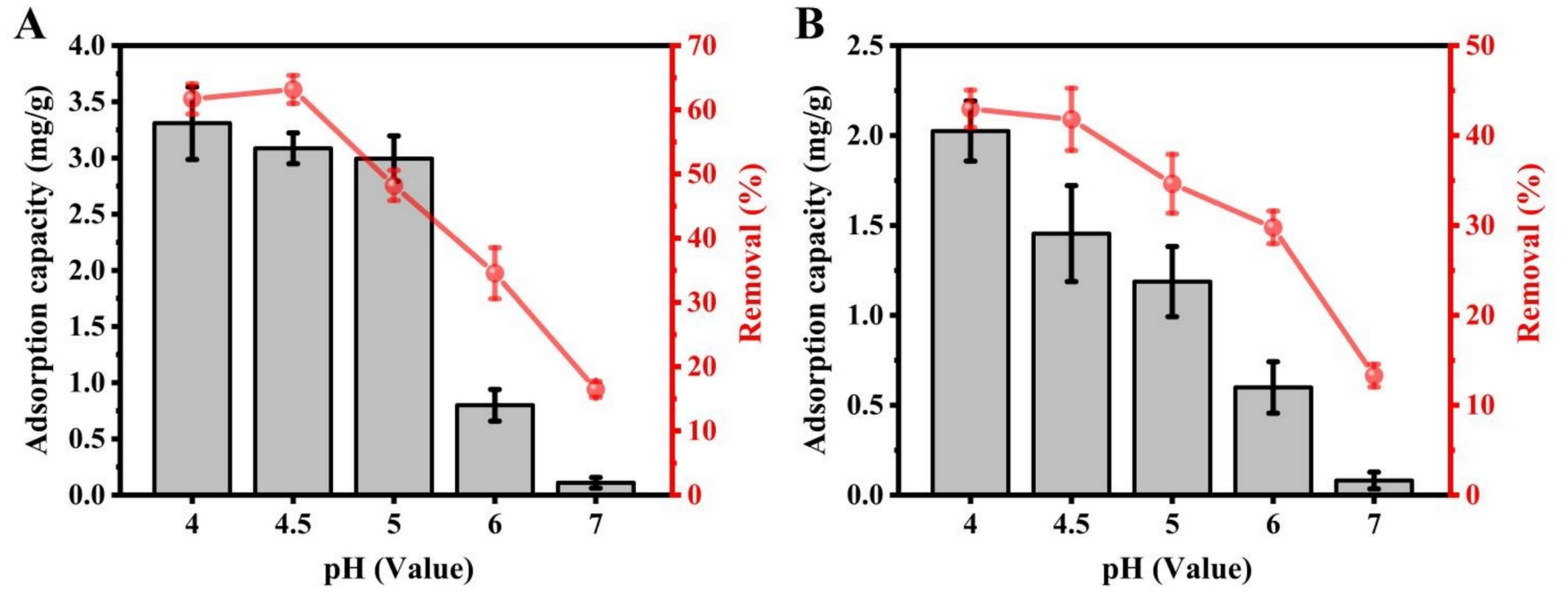
Effect of the pH on Cd uptake and removal by strains C9 and C27. **(A)** Strain C9, **(B)** Strain C27.

To determine the influence of pH on Cd^2+^ adsorption by strains C9 and C27, the pH of the culture medium was adjusted to 4, 4.5, 5, 6, and 7. According to the ICP-MS results, the amount of Cd^2+^ adsorbed by strain C9 was 3.31 mg/g dry weight (DW), 3.09 mg/g DW, 2.99 mg/g DW, 0.80 mg/g DW, and 0.11 mg/g DW, with corresponding removal rates of 61.69, 63.18, 48.23, 34.55, and 16.47%, respectively. For strain C27, the adsorbed amounts were 2.02 mg/g DW, 1.45 mg/g DW, 1.19 mg/g DW, 0.60 mg/g DW, and 0.08 mg/g DW, with removal rates of 42.96, 41.79, 34.63, 29.78, and 13.29%, respectively (Figure 3). In conclusion, the maximum Cd^2+^ adsorption for both strains occurred at pH levels between 4 and 4.5.

##### 3.2.1.2 Effect of incubation on adsorption

**Figure 4:**
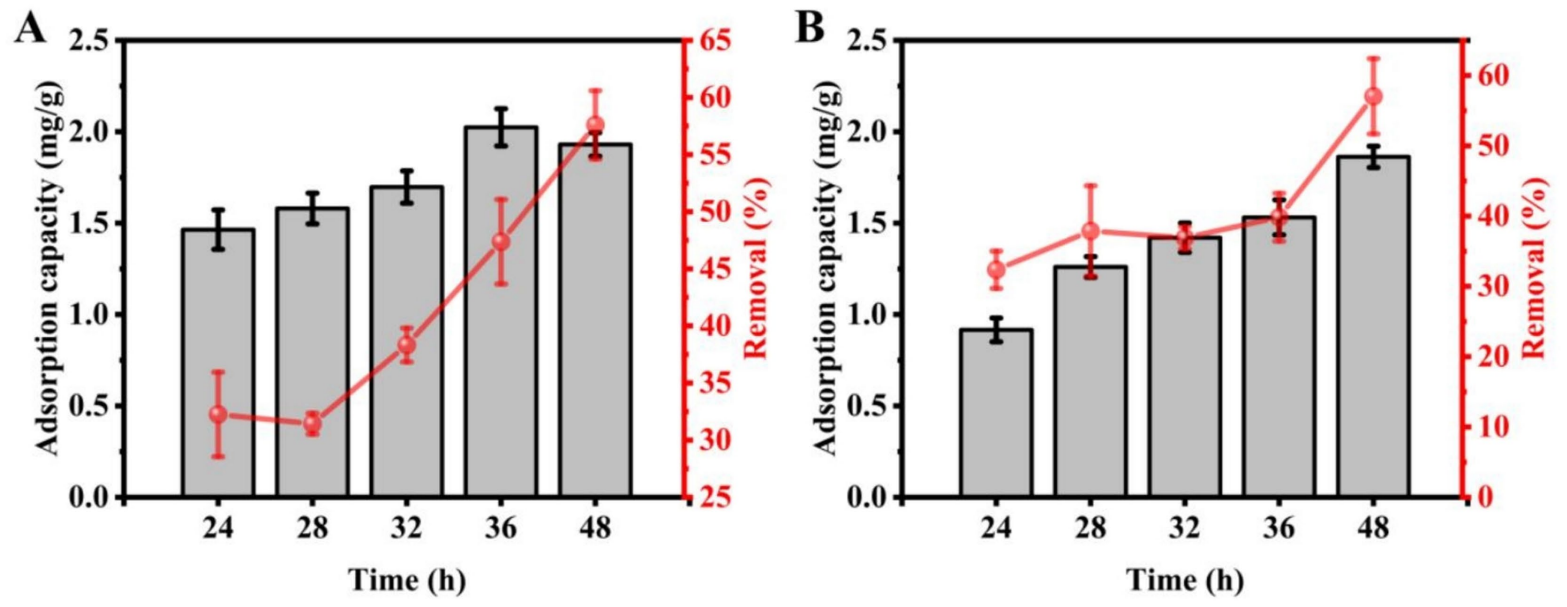
Effect of adsorption time on cadmium absorption by strains C9 and C27. **(A)** Strain C9, **(B)** Strain C27.

To assess the impact of adsorption time on Cd^2+^ uptake by strains C9 and C27, adsorption durations of 24, 28, 32, 36, and 48 h were tested. ICP-MS data indicated that the amount of Cd^2+^ adsorbed by strain C9 was 1.46 mg/g, 1.58 mg/g, 1.70 mg/g, 2.02 mg/g, and 1.93 mg/g at the respective time points, with corresponding removal rates of 32.25, 31.44, 38.33, 47.37, and 57.60%. Similarly, strain C27 adsorbed 0.92 mg/g, 1.26 mg/g, 1.42 mg/g, 1.53 mg/g, and 1.86 mg/g at the respective time points, with removal rates of 32.37, 37.88, 36.91, 39.84, and 57.05% (Figure 4). Strain C9 achieved peak Cd^2+^ uptake at 36 h, with the highest removal rate observed at 48 h. Conversely, strain C27 exhibited maximum Cd^2+^ adsorption and removal rates at 48 h. In conclusion, the optimal adsorption time was found to be 36 h for strain C9, and 48 h for strain C27.

##### 3.2.1.3 Resistance to other heavy metals

Strains C9 and C27 are capable of proliferating in media with elevated concentrations of cadmium (Cd), Zinc (Zn), and thallium (Tl). Zn is an essential trace nutrient for bacteria, whereas Cd and Tl are toxic and potentially lethal. Table 1 presents the minimum inhibitory concentrations (MICs) for Zn, Cd, and Tl for strains C9 and C27. It is evident that both strains exhibit resistance to all three metals, with strain C9 demonstrating higher resistance than strain C27. The resistance profile for both strains follows the order Zn > Cd > Tl.

**Table 1:** MIC (mg/L) of various heavy metals.

Metal	Strain C9	Strain C27
Zn	1,600	1,500
Cd	550	550
Tl	500	400

### 3.3 Physiological and biochemical analysis of isolated strains

#### 3.3.1 Siderophore production

**Figure 5:**
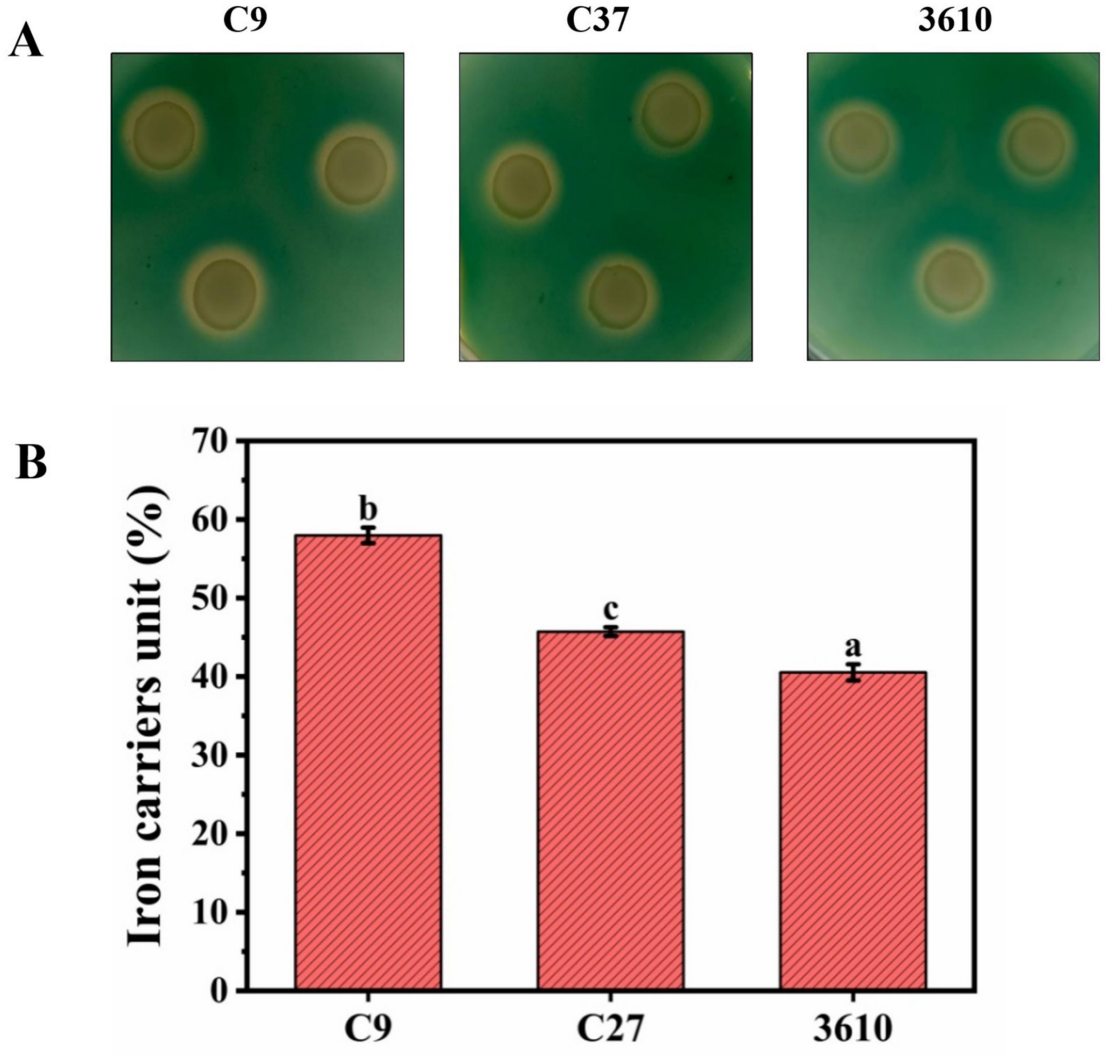
**(A)** Experimental results of qualitative analysis on siderophores of three strains. **(B)** Quantitative analysis of iron carriers in Cd-resistant strains C9, C27, and model bacterium 3,610.

Strain C9, strain C27, and the model 3,610 all produced orange-yellow halos, indicating that all three strains have the ability to synthesize siderophores (Figure 5A). The active units of siderophores produced by strains C9, C27, and the model strain 3,610 were measured as 57.96, 45.72, and 40.53, respectively (Figure 5B). The siderophore production capability ranked as follows: C9 > C27 > 3,610, with statistically significant differences observed. Therefore, the siderophores-producing ability of strains C9 and C27 could be a contributing factor to their tolerance to Cd.

#### 3.3.2 Remaining physiological and biochemical analyses

##### 3.3.2.1 Indole-3-acetic acid (IAA) production

The results showed that strains C9, C27, and strain 3,610 were capable of producing IAA, with yields of 44.37 mg/L, 49.82 mg/L, and 65.77 mg/L, respectively, exhibiting significant variability (Supplementary Figure S2). These results indicated that IAA production is not the primary reason for the Cd resistance of strains C9 and C27.

##### 3.3.2.2 Biofilm formation

In liquid medium, strains C9, C27, and 3,610 all initially formed biofilms after 24 h of incubation. After 48 h, the model bacterium 3,610 had developed a wrinkled pellicle on the liquid surface, while strains C9 and C27 developed wrinkled pellicles on their surfaces after 72 h of incubation. This indicates that the biofilm-forming ability of the model bacterium 3,610 is stronger than those of strains C9 and C27 (Supplementary Figure S3). On solid medium, all three strains were capable of colony biofilm formation, with the model bacterium 3,610 showing the strongest colony biofilm production after 72 h of incubation (Supplementary Figure S4). Although biofilms enhance survival in stressful conditions, they do not appear to be the primary mechanism underlying cadmium resistance in these strains.

##### 3.3.2.3 Salt tolerance and starch hydrolysis by isolates

Strains C9 and C27 exhibited growth on solid media with NaCl concentrations ranging from 5 to 13%, indicating that both strains possess salt tolerance (Supplementary Figure S5). However, a high concentration of NaCl led to reduced growth in strain C9, while having a minor effect on the growth of strains C27 and C3610. This salt tolerance characteristic enhances their potential for application in saline soils, where they could assist in promoting plant growth.

The ability of the strains to hydrolyze starch can be determined by the presence of a transparent halo around the colonies on a starch agar medium. Supplementary Figure S6 shows that transparent halos appeared around the colonies of Cd-resistant strains C9, C27, and model bacterium 3,610, indicating their ability to hydrolyze starch.

##### 3.3.2.4 Citrate utilization experiment

The citrate utilization test was conducted to assess the ability of the strain to utilize citrate as a chelating agent for Cd tolerance. Strains C9, C27, and the model bacterium 3,610 were inoculated onto citrate-containing solid medium, and the color change of the colonies was observed. Supplementary Figure S7 illustrates that all Cd-resistant strains (C9, C27, and model bacterium 3,610) exhibited a blue coloration during growth, indicating their ability to degrade and utilize citrate as a carbon source essential for growth. Furthermore, strain C9 demonstrated a more pronounced rate of citrate utilization than strain C27.

##### 3.3.2.5 Ammonia production experiment

To assess whether the strains could fix nitrogen, the color change in the peptone water medium was monitored after the addition of Garner’s reagent to detect ammonia production. In the test tubes, strains C9, C27, and the model bacterium 3,610 all exhibited yellow precipitation, with strain C9 showing a significantly stronger effect compared to strain C27 and the model bacterium 3,610. This suggests that strain C9 has a higher capacity for ammonia production (Supplementary Figure S8).

Strains C9 and C27, identified as *Bacillus cereus*, exhibited high Cd resistance and removal efficiency under optimal conditions. Their resistance to other heavy metals, physiological versatility, and biochemical capabilities underscores their potential for bioremediation applications. These findings contribute valuable insights into the mechanisms of microbial Cd resistance and the development of effective strategies for remediating heavy metal-contaminated soils.

## 4 Discussion

In this study, *Bacillus cereus* strains C9 and C27 were identified as highly resistant to cadmium and demonstrated their significant potential for Cd adsorption and removal. The genus *Bacillus* is one of the predominant bacterial genera found in soil, and several species within this genus have been reported in diverse ecological niches (Saxena et al., 2020; Zada et al., 2021; Qian et al., 2022). The findings align with previous research that highlights the potential of *Bacillus* species in remediating heavy metal contamination through multiple mechanisms, including adsorption, metal binding, and siderophore production (Wróbel et al., 2023; Alotaibi et al., 2021). The mechanisms underlying the cadmium resistance of these strains are multifaceted. Renu et al. (2022) reported that cell wall modifications also play a crucial role. The cell walls of *Bacillus cereus* strains C9 and C27 might be modified to bind cadmium ions more effectively, preventing them from entering the cytoplasm and causing damage.

Strains C9 and C27 achieved peak Cd adsorption at moderate concentrations (70 μM) and acidic pH (4.5), with strain C9 demonstrating a slightly higher adsorption efficiency than strain C27. These results are consistent with previous studies, which reported optimal Cd adsorption for *Bacillus cereus* in acidic conditions (pH 4.5–5.0), due to the protonation of functional groups on the bacterial surface, enhancing electrostatic attraction to Cd ions (Todorova et al., 2019). However, adsorption efficiency declined at higher concentrations (90 μM), possibly due to site saturation and competitive interactions, a phenomenon also noted by Zhao et al. (2017). Compared to *Bacillus megaterium*, which reached a maximum Cd adsorption of 1.3 mg/g (Fang et al., 2016), strain C9 exhibited higher adsorption (1.8 mg/g), highlighting its potential as a more effective bioremediator. The adsorption kinetics also mirrored findings in *Bacillus thuringiensis*, where extended incubation improved Cd removal rates up to 36–48 h, as reported by Çolak et al. (2011). Moreover, the decline in adsorption efficiency could also be related to the cadmium resistance mechanisms of the strains. When cadmium concentrations are extremely high, the strain may activate additional detoxification mechanisms such as the production of stress proteins (Khan et al., 2016). These proteins can bind to cadmium ions in the cytoplasm, reducing their free concentration and toxicity. This process, however, might divert the strain’s resources from the adsorption process, leading to a decrease in adsorption efficiency. Both strains displayed a metal resistance hierarchy with Zn > Cd > Tl, and strain C9 showed remarkable Zn tolerance up to 1,600 mg/L, exceeding the Zn tolerance reported for *Bacillus cereus* (800 mg/L) (Fang et al., 2016), indicating the strains’ resilience under heavy metal stress. Similar resistance patterns were observed in *Bacillus* sp., which adapted to multiple metals through mechanisms such as efflux systems and extracellular polymeric substances (EES) (Nair et al., 1993). Microorganisms can rapidly interact with extracellular metals and play an important role in metal tolerance through metal dispersion, transport, immobilization, and transformation (Lu et al., 2023).

The effect of initial pH on cadmium uptake by strains C9 and C27 was studied within the pH range of 4.0 to 7.0. Cd uptake significantly decreased as pH increased, with strain C9’s uptake falling from 3.31 mg/g to 0.11 mg/g and strain C27’s from 2.02 mg/g to 0.08 mg/g. The maximum Cd removal for both strains was observed at pH 4.5, suggesting it as the optimal pH for biosorption. This trend is consistent with previous research showing the best metal removal efficiency in similar pH ranges (4.5–5.0) (Todorova et al., 2019; Valero et al., 2003). The reduction in Cd uptake with increasing pH is attributed to the competition between metal ions and hydroxide ions (OH^−^), which can result in the precipitation of metal hydroxides and decrease the availability of Cd. At lower pH, the increased concentration of hydrogen ions (H^+^) enhances metal ion binding, improving biosorption (Wen et al., 2018; Pan et al., 2005; Çolak et al., 2011). These results highlight the importance of optimizing pH for effective bioremediation applications, with strain C9 showing superior Cd removal at pH 4.5. Meanwhile, by integrating the previous studies, it was found that the strains had superior cadmium removal ability when the pH was 4–5 (Supplementary Table S1) (Chi et al., 2024; Sandrin and Maier, 2002). Further research is needed to explore the mechanisms behind this enhanced biosorption.

The impact of adsorption time on cadmium removal by strains C9 and C27 revealed a significant growth in Cd uptake with time, particularly for strain C27. Cd adsorption by strain C9 increased sharply initially, reaching a peak at 36 h, after which it stabilized, signifying that the adsorption equilibrium had been achieved. This pattern, with rapid initial adsorption followed by stabilization, is typical of biosorption processes, as seen in other studies like those on Pb^2+^ by *Bacillus* species (Çolak et al., 2011). The initial rapid uptake is driven by the availability of binding sites, while the stabilization reflects the exhaustion of these sites and the attainment of equilibrium.

Strains C9 and C27 exhibited substantially greater siderophore production compared to the model bacterium *Bacillus subtilis* 3,610, implying that siderophores play a critical role in Cd resistance by complexing metal ions and diminishing their bioavailability (Roy et al., 2022). Siderophore activity has been similarly implicated in heavy metal tolerance in *Pseudomonas fluorescens*, where increased siderophore production enhanced Cd adsorption efficiency (Khan et al., 2020). The higher siderophore activity observed in strain C9 (57.96 SU) compared to strain C27 (45.72 SU) likely explains its superior Cd tolerance. Both strains displayed traits beneficial for plant growth and soil remediation, including salt tolerance, ammonia production, starch hydrolysis, and citrate utilization. Their capacity to thrive in saline conditions (up to 13% NaCl) indicates their suitability for remediating Cd-contaminated saline soils, a feature shared with *Enterobacter* sp., as noted by Pramanik et al. (2018). Moreover, the ammonia production of strain C9 was significantly higher than strain C27 and *Bacillus subtilis*, indicating its potential to augment soil fertility in contaminated areas.

While many studies have reported Cd resistance and adsorption capabilities in *Bacillus* species, strains C9 and C27 exhibited unique advantages, including higher adsorption capacities, significant siderophore activity, and robust salt tolerance. For instance, Junpradit et al. (2021) reported Cd removal efficiencies of up to 24% in *E. cloacae*, which is lower than the maximum removal rate of 32.27% observed for strain C27. Moreover, *Bacillus cereus* strains studied by Zhou et al. (2024) showed comparable resistance to Cd but lacked the broad-spectrum resistance to Zn and Tl observed in the current study. These results highlight the competitive edge of strains C9 and C27 for application in bioremediation, particularly in multi-metal contaminated environments. Additionally, their physiological versatility makes them suitable for diverse ecological conditions, further expanding their practical utility.

The Cd tolerance observed in strains C9 and C27 can be attributed to a combination of factors, including surface adsorption, siderophore production, and biofilm formation. While biofilms provide a protective barrier against Cd toxicity, their contribution was less pronounced in these strains compared to siderophores. This aligns with findings by Hao et al. (2013), who emphasized siderophores as a primary resistance mechanism in heavy metal-tolerant bacteria. The findings of this study underscore the potential of *Bacillus cereus* strains C9 and C27 for sustainable bioremediation of Cd-contaminated soils. Future research should focus on unraveling the genetic and molecular mechanisms underlying their metal resistance and optimizing their application in field-scale remediation projects. Incorporating these strains into phytoremediation systems could further enhance their efficacy by leveraging plant-microbe interactions.

## 5 Conclusion

Strains C9 and C27, both of which exhibit high Cd resistance, were isolated. Based on the characterization of these strains and comparisons of their 16S rDNA sequences, both strains were identified as *Bacillus cereus.* The removal of Cd^2+^ by strains C9 and C27 occurred at a Cd^2+^ concentration of 70 μM, at 26.75 and 32.27%, respectively. The optimal initial concentrations for Cd^2+^ adsorption were 70 μM and 90 μM. According to the growth curves based on adsorption time, the optimal adsorption time was 36 h for strain C9 and 48 h for strain C27.

In addition to Cd resistance, strains C9 and C27 also demonstrated tolerance to other metals such as Zn and Tl on solid media plates. The order of resistance of these strains to metals was found to be Zn > Cd > Tl. Both the Cd-resistant strains exhibited high salt tolerance, starch hydrolysis ability, citrate utilization, and ammonia production. Strains C9, C27, and model bacterium 3,610 were capable of producing IAA, biofilms, and iron carriers. However, strains C9 and C27 were more adept at producing iron carriers than the model bacterium 3,610, with C9 being even more proficient than C27. It was hypothesized that the production of iron carriers might be the reason for the Cd tolerance of strains C9 and C27.

## Data Availability

The datasets presented in this study can be found in online repositories. The names of the repository/repositories and accession number(s) can be found in the article/Supplementary material.
