# Supplementary material for: Isolation and characterization of cadmium-resistant Bacillus cereus strains from Cd-contaminated mining areas for potential bioremediation applications
Source: Front Microbiol. 2025 Feb 12;16:1550830. doi: 10.3389/fmicb.2025.1550830 (PMC11861182; doi:10.3389/fmicb.2025.1550830)
Supplement: Supplementary file 1 [file Data_Sheet_1.PDF]

Supporting information

Text1 Sequence results

Sequence results for the *Bacillus cereus* strain C9

TCGAAGCGAAATGGGATTAAGAGCTTGCTCTTATGAAGTTAGCGGCGGACGGGTGA
GTAACACGTGGGTAACCTGCCCATAAGACTGGGATAACTCCGGGAAACCGGGGCTAAT
ACCGGATAAYATTTTGAACCGCATGGTTCGAAATTGAAAGGCGGCTTCGGCTGTCACTTA
TGGATGGACCCGCGTCGCATTAGCTAGTTGGTGAGGTAACGGCTCACCAAGGCAACGAT
GCGTAGCCGACCTGAGAGGGTGATCGGCCACACTGGGACTGAGACACGGCCCAGACTC
CTACGGGAGGCAGCAGTAGGGAATCTTCCGCAATGGACGAAAGTCTGACGGAGCAACG
CCGCGTGAGTGATGAAGGCTTTCGGGTCGTAAAACTCTGTTGTTAGGGAAGAACAAGT
GCTAGTTGAATAAGCTGGCACCTTGACGGTACCTAACCAGAAAGCCACGGCTAACTACG
TGCCAGCAGCCGCGGTAATACGTAGGTGGCAAGCGTTATCCGGAATTATTGGGCGTAAA
GCGCGCGCAGGTGGTTTCTTAAGTCTGATGTGAAAGCCCACGGCTCAACCGTGGAGGG
TCATTGGAAACTGGGAGACTTGAGTGCAGAAGAGGAAAGTGGAATTCCATGTGTAGCG
GTGAAATGCGTAGAGATATGGAGGAACACCAGTGGCGAAGGCGACTTTCTGGTCTGTAA
CTGACACTGAGGCGCGAAAGCGTGGGGAGCAAACAGGATTAGATACCCTGGTAGTCCA
CGCCGTAAACGATGAGTGCTAAGTGTTAGAGGGTTTCCGCCCTTTAGTGCTGAAGTTAA
CGCATTAAGCACTCCGCCTGGGGAGTACGGCCGCAAGGCTGAAACTCAAAGGAATTGA
CGGGGGCCCGCACAAGCGGTGGAGCATGTGGTTTAATTCGAAGCAACGCGAAGAACCT
TACCAGGTCTTGACATCCTCTGAAAACCCTAGAGATAGGGCTTCTCCTTCGGGAGCAGA
GTGACAGGTGGTGCATGGTTGTCGTCAGCTCGTGTCGTGAGATGTTGGGTTAAGTCCCG
CAACGAGCGCAACCCTTGATCTTAGTTGCCATCATTAAGTTGGGCACTCTAAGGTGACTG
CCGGTGACAAACCGGAGGAAGGTGGGGATGACGTCAAATCATCATGCCCCTTATGACCT
GGGCTACACACGTGCTACAATGGACGGTACAAAGAGCTGCAAGACCGCGAGGTGGAGC
TAATCTCATAAAACCGTTCTCAGTTCGGATTGTAGGCTGCAACTCGCCTACATGAAGCTG
GAATCGCTAGTAATCGCGGATCAGCATGCCGCGGTGAATACGTTCCCGGGCCTTGTACAC
ACCGCCCGTCACACCACGAGAGTTTGTAACACCCGAAGTCGGTGGGGTAACCTTTTGG
AGCCAGCCGCC

Sequence results for the *Bacillus cereus* strain C27

TGCAAGTCGAGCGAATGGATTAAGAGCTTGCTCTTATGAAGTTAGCGGCGGACGGG
TGAGTAACACGTGGGTAACCTGCCCATAAGACTGGGATAACTCCGGGAAACCGGGGCTA
ATACCGGATAAYATTTTGAACYGCATGGTTCGAAATTGAAAGGCGGCTTCGGCTGTCACT
TATGGATGGACCCGCGTCGCATTAGCTAGTTGGTGAGGTAACGGCTCACCAAGGCAACG
ATGCGTAGCCGACCTGAGAGGGTGATCGGCCACACTGGGACTGAGACACGGCCCAGAC
TCCTACGGGAGGCAGCAGTAGGGAATCTTCCGCAATGGACGAAAGTCTGACGGAGCAA
CGCCGCGTGAGTGATGAAGGCTTTCGGGTCGTAAAACTCTGTTGTTAGGGAAGAACAA
GTGCTAGTTGAATAAGCTGGCACCTTGACGGTACCTAACCAGAAAGCCACGGCTAACTA
CGTGCCAGCAGCCGCGGTAATACGTAGGTGGCAAGCGTTATCCGGAATTATTGGGCGTA
AAGCGCGCGCAGGTGGTTTCTTAAGTCTGATGTGAAAGCCCACGGCTCAACCGTGGAG
GGTCATTGGAAACTGGGAGACTTGAGTGCAGAAGAGGAAAGTGGAATTCCATGTGTAG
CGGTGAAATGCGTAGAGATATGGAGGAACACCAGTGGCGAAGGCGACTTTCTGGTCTGT
AACTGACACTGAGGCGCGAAAGCGTGGGGAGCAAACAGGATTAGATACCCTGGTAGTC
CACGCCGTAAACGATGAGTGCTAAGTGTTAGAGGGTTTCCGCCCTTTAGTGCTGAAGTT
AACGCATTAAGCACTCCGCCTGGGGAGTACGGCCGCAAGGCTGAAACTCAAAGGAATT
GACGGGGGCCCGCACAAGCGGTGGAGCATGTGGTTTAATTCGAAGCAACGCGAAGAAC
CTTACCAGGTCTTGACATCCTCTGAAAACCCTAGAGATAGGGCTTCTCCTTCGGGAGCA
GAGTGACAGGTGGTGCATGGTTGTCGTCAGCTCGTGTCGTGAGATGTTGGGTTAAGTCC
CGCAACGAGCGCAACCCTTGATCTTAGTTGCCATCATTAAGTTGGGCACTCTAAGGTGA
CTGCCGGTGACAAACCGGAGGAAGGTGGGGATGACGTCAAATCATCATGCCCCTTATGA
CCTGGGCTACACACGTGCTACAATGGACGGTACAAAGAGCTGCAAGACCGCGAGGTGG
AGCTAATCTCATAAAACCGTTCTCAGTTCGGATTGTAGGCTGCAACTCGCCTACATGAAG
CTGGAATCGCTAGTAATCGCGGATCAGCATGCCGCGGTGAATACGTTCCCGGGCCTTGTA
CACACCGCCCGTCACACCACGAGAGTTTGTAACACCCGAAGTCGGTGGGGTAACCTTTT
TGGAGCCAGCCGCCTAA

Sequence results for Bacterial universal primer 27F

5′ - AGAGTTTGATCCTGGCTCAG - 3′

Sequence results for Bacterial universal primer 1492R

5′ - GGTTACCTTGTTACGACTT - 3′

Text2 Additional images

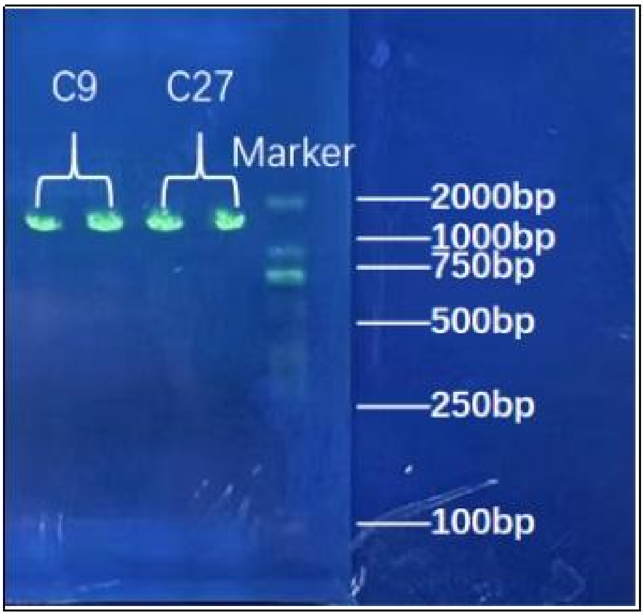

Fig. S1 Electrophoresis results of Cd-resistant strains C9 and C27.

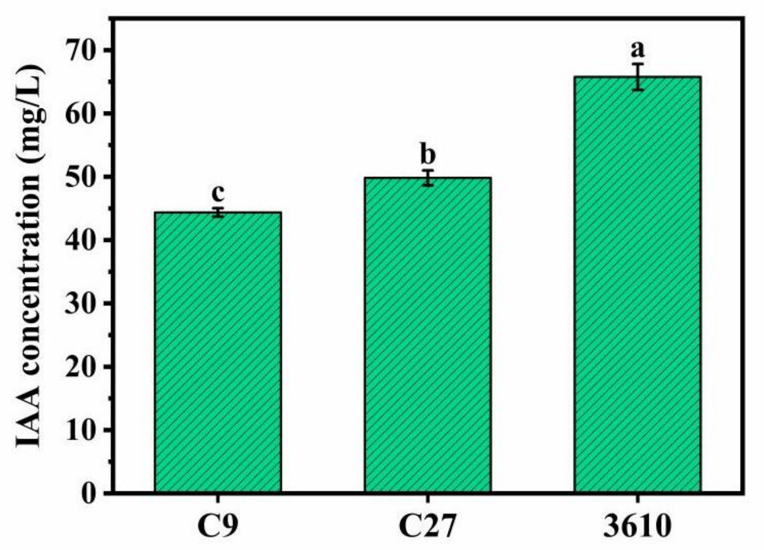

Fig. S2 Analysis of IAA production by strains

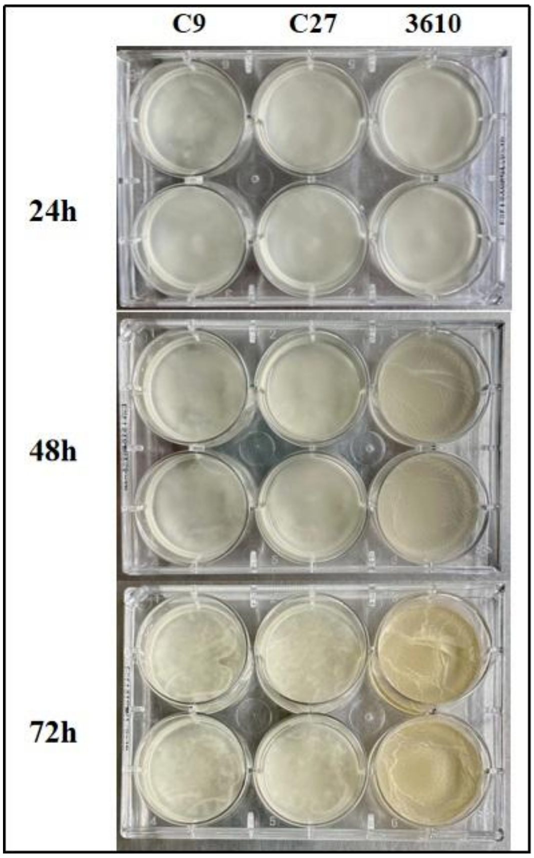

Fig. S3 Growth of Cd-resistant strains C9, C27 and model strain 3610 in liquid medium at different time periods.

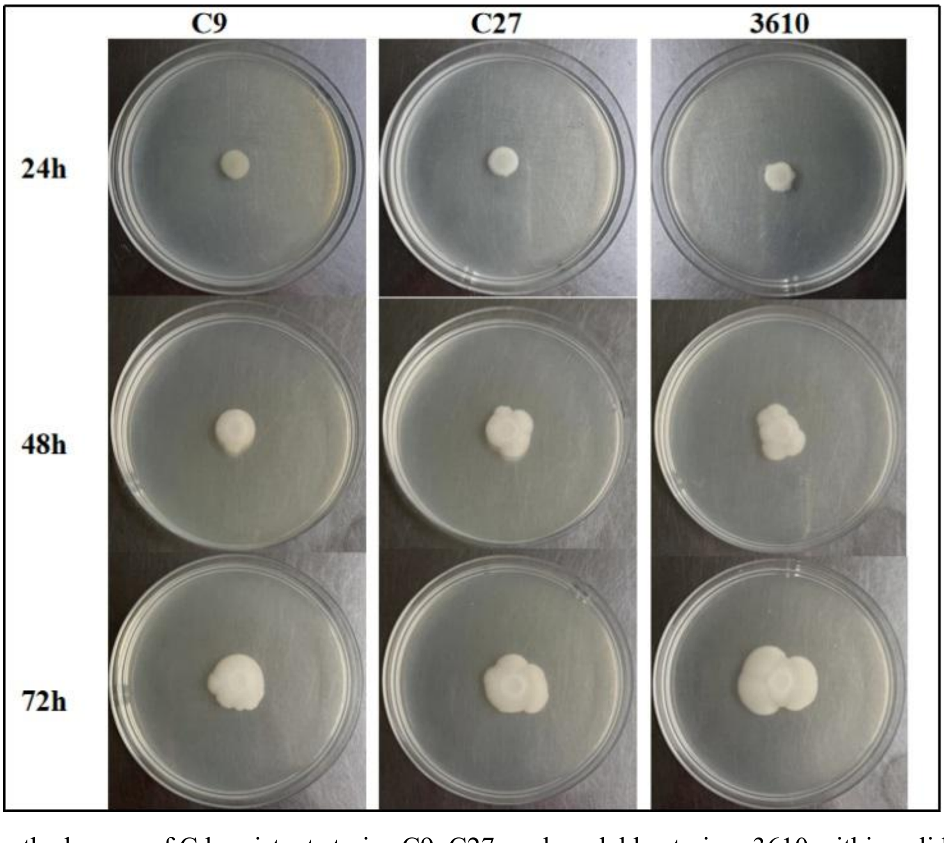

Fig. S4 Growth changes of Cd-resistant strains C9, C27, and model bacterium 3610 within solid biofilms at different time periods.

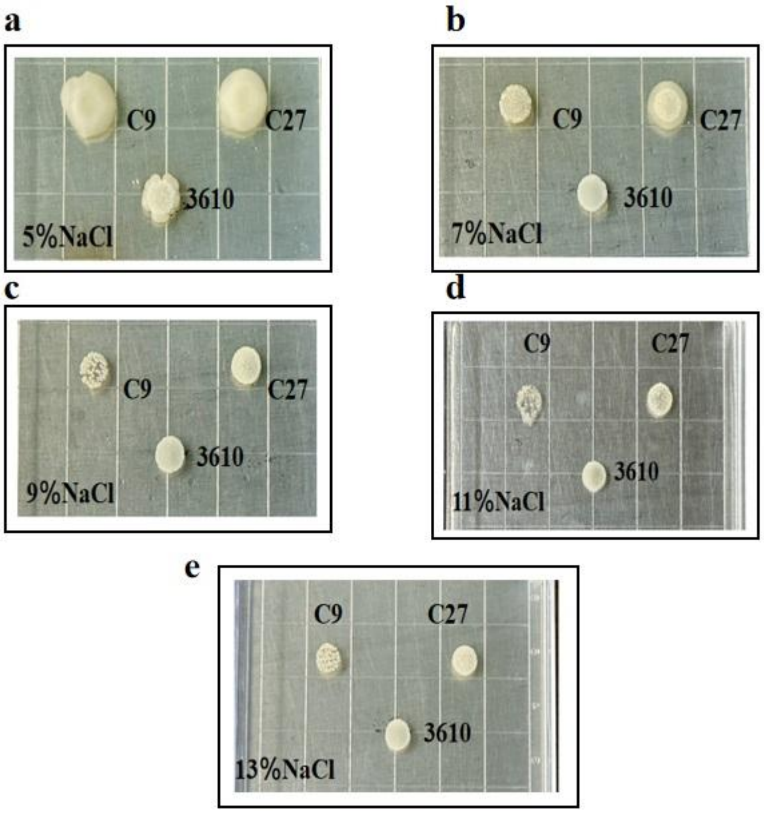

Fig. S5 Experimental results for Cd-resistant strains C9, C27 and model bacterium 3610.

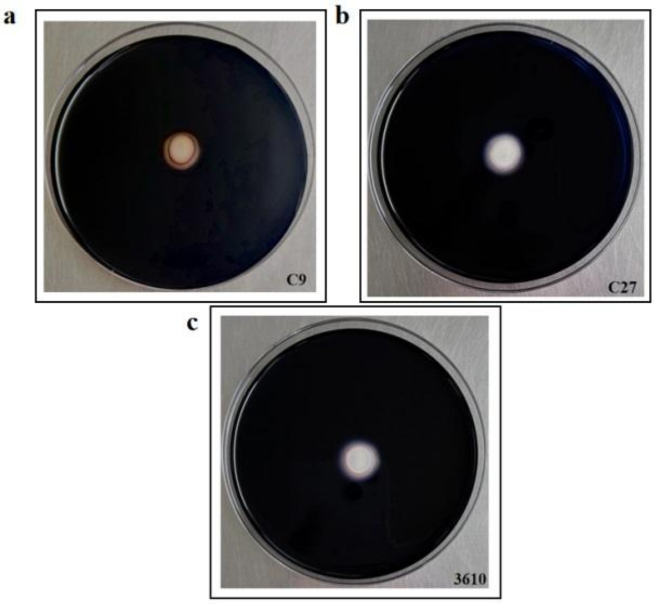

Fig. S6 Hydrolysis of starch by Cd-resistant strains C9, C27 and model bacterium 3610

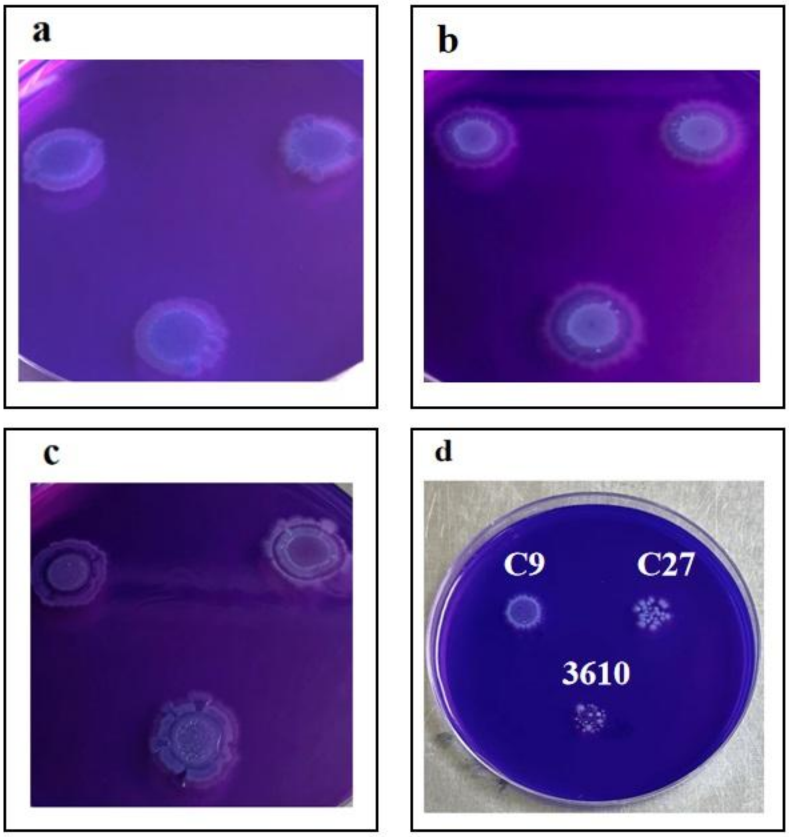

Fig. S7 Utilization results of citrate among strains C9, C27, and model bacterium.

Note:(a) Experimental results of citrate utilization by strain C9, (b) Experimental results of citrate utilization by strain C27, (c) Experimental results of citrate utilization by model bacterium 3610, (d) Comparative results of three strains of bacteria

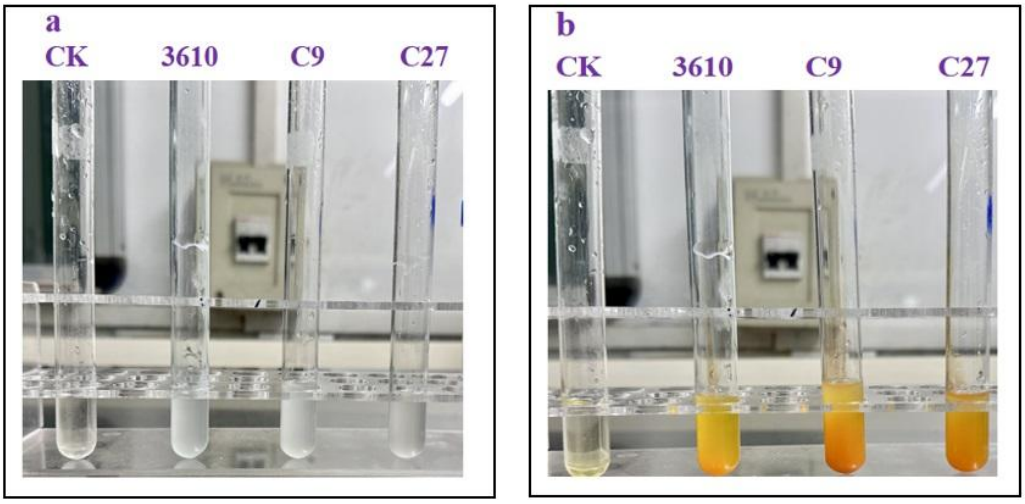

Fig. S8 Experimental results of ammonia production by strains C9, C27, and model bacterium 3610.
Note: (a) Before the addition of Nath's reagent, (b) After the addition of Nath's reagent

Table S1 Cd removal by strains analyzed in conjunction with previous studies

Strain	Biosorption parameters			Removal (%)	References
	pH	Cd concentration	adsorption time		
Bacillus cereus C9	4.5	50 μM	24 h	63.18%	This study
Bacillus cereus C27	4	50 μM	24 h	42.96%	This study
Klebsiella sp. AW2	5	10 mg/L	4 h	40.99%	(Chi et al., 2024)
Burkholderia sp.	4	334 μM	40 h	67.61%	(Sandrin et al., 2002)
